# Ex Vivo Osteogenesis Induced by Calcium Silicate-Based Cement Extracts

**DOI:** 10.3390/jfb14060314

**Published:** 2023-06-07

**Authors:** Gabriel Kato, Rita Araújo, Cláudia Rodrigues, Pedro Sousa Gomes, Liliana Grenho, Maria Helena Fernandes

**Affiliations:** 1 Laboratory for Bone Metabolism and Regeneration, Faculty of Dental Medicine, University of Porto, 4200-393 Porto, Portugalmhfernandes@fmd.up.pt (M.H.F.); 2 LAQV/Requimte, University of Porto, 4100-007 Porto, Portugal; 3 Faculty of Dental Medicine, University of Porto, 4200-393 Porto, Portugal

**Keywords:** fast-setting cements, Biodentine^TM^, TotalFill^®^, ProRoot^®^ MTA, bone formation, ex vivo model

## Abstract

Calcium silicate-based cements are used in a variety of clinical conditions affecting the pulp tissue, relying on their inductive effect on tissue mineralization. This work aimed to evaluate the biological response of calcium silicate-based cements with distinct properties—the fast-setting Biodentine™ and TotalFill^®^ BC RRM™ Fast Putty, and the classical slow-setting ProRoot^®^ MTA, in an ex vivo model of bone development. Briefly, eleven-day-old embryonic chick femurs were cultured for 10 days in organotypic conditions, being exposed to the set cements’ eluates and, at the end of the culture period, evaluated for osteogenesis/bone formation by combining microtomographic analysis and histological histomorphometric assessment. ProRoot^®^ MTA and TotalFill^®^ extracts presented similar levels of calcium ions, although significantly lower than those released from Biodentine^TM^. All extracts increased the osteogenesis/tissue mineralization, assayed by microtomographic (BV/TV) and histomorphometric (% of mineralized area; % of total collagen area, and % of mature collagen area) indexes, although displaying distinct dose-dependent patterns and quantitative values. The fast-setting cements displayed better performance than that of ProRoot^®^ MTA, with Biodentine^TM^ presenting the best performance, within the assayed experimental model.

## 1. Introduction

Calcium silicate-based materials have a wide range of applications, as reported in a recent comprehensive bibliometric analysis of the published research literature on these materials, over the past three decades, which identified three major clusters, i.e., dentistry, bone tissue, and bioactivity [1]. In dentistry, calcium silicate-based cements (CSCs) comprise a group of endodontic repair materials that react and set in the presence of water. The setting behavior includes first, a hydration reaction of tricalcium and dicalcium silicates leading to the formation of calcium hydroxide and following, the precipitation of calcium phosphate, providing these materials with bioactivity and biocompatibility favoring hard tissue mineralization in repair, regeneration and formation events [2,3,4,5,6]. Accordingly, associated clinical applications include root canal obturation, retro-filling in periapical surgeries, and repair of perforation and resorptive defects [1,6]. Upon the cement application, in the moist biological environment, calcium, silicate, and hydroxide ions are released from the material during and after the setting reaction, with all these ions contributing to provide a positive biocompatibility and osteoinductive/osteoconductive environment favoring tissue mineralization [6,7,8,9,10].

The gold standard material has been mineral trioxide aggregate (MTA), although it presents some drawbacks and manipulation challenges. The first MTA formulation, introduced by Dr. Mahmoud Torabinejad, is a Portland-based material, composed of tricalcium and dicalcium silicate, calcium carbonate, calcium sulfate, and calcium aluminate, with bismuth oxide as the radiopacifying agent [4,5]. The material has been extensively studied before being commercialized as ProRoot^®^ MTA (Dentsply, Tulsa, OK, USA) in 1998, to be used as a root repair material. It is presented as a powder–liquid material to be manually mixed, with a long initial setting time, ~around 4 h [4]. New formulations for CSCs have been approached with modified composition—as the five mineral oxides (5MO), added micro- and macro-compounds that aim to enhance both physicochemical and biological properties, for improved clinical outcomes [3,11,12]. Biodentine™ (Septodont, Saint-Maur-des-Fossés, France) was one of the first formulations proposed to replace MTA for repair and regenerative endodontics. It contains tricalcium and dicalcium silicates, calcium carbonate, iron oxide, zirconium oxide as an opacifier, and the liquid phase is composed of calcium chloride and a hydrosoluble polymer [13]. The initial setting time is around 12 min after mixing, and is indicated for a branch of repair procedures, from pulp capping, furcal perforation repair, and root-end filling [13,14,15]. Additionally, some of the current bioactive CSCs have been developed as pre-mixed materials with putty or paste consistency. These include EndoSequence Root Repair Material RRM (Brasseler, Savannah, USA) and TotalFill^®^ BC RRM™ Fast Putty (FKG Dentaire SA, La Chaux-de-Fonds, Switzerland), which present a fast-setting and bulk consistency [6,16,17]. Overall, available clinical research tends to support successful treatment outcomes with calcium silicate-based cements [18,19]. However, compared to ProRoot^®^ MTA, with an established clinical use and subjected to a multitude of studies [1,3,5], the fast-setting materials, namely the pre-mixed formulations, have been used clinically for a shorter time [20]. Thus, long-term comparative clinical outcomes of slow- and fast-setting cements are largely missing [13,19].

Upon application, the fresh cement is exposed to the surrounding environment coming into direct contact with blood or tissue fluids, and in this context, the setting-time profile might assume particular relevance in the cement physicochemical features and eluates’ kinetics, affecting the cellular and tissue response [21,22]. This is accounted for by some studies reporting that early contamination with blood or tissue fluids can disturb proper setting and compromise the cement mechanical properties, namely the compression strength and surface microhardness, of the slow-setting ProRoot^®^ MTA [22,23,24,25]. This turns out to be relevant as often, the actual clinical conditions are suboptimal and far from the ideal environment for the proper setting of CSCs. In this context, expectedly, the fast-setting cements would be associated with lower susceptibility to detrimental local conditions, as previously suggested in a comparative study between ProRoot^®^ MTA and Retro MTA (BioMTA, Seoul, Republic of Korea), a fast-setting material [26].

In conditions demanding the formation of mineralized tissues, CSCs affect significantly and distinctly the behavior of different local cells—e.g., apical papilla cells, periodontal ligament cells, endothelial cells, bone marrow stromal cells, and bone cells. These populations present site specificity and are engaged in variable stages of proliferative/differentiation commitment, being distinctively affected by the niche microenvironment. In this frame, the osteogenic induction of osteoblastic lineage cells is a key event for the desired tissue mineralization in reparative endodontics. In vitro studies have established the relevance of the release of calcium and silicon ions to this process [9,27]. In particular, the relevance of calcium release dynamics in the mineralization outcomes of these materials is well documented in several in vitro studies involving different osteogenic precursor cells [28,29,30], owing to the extracellular and intracellular role of Ca^2+^ in a large variety of key cellular pathways and signaling mechanisms [31]. Due to the possibility of having a strictly monitored physiochemical environment of culture conditions, the in vitro models have contributed decisively to elucidate the complex network of signaling pathways, molecules, and transcriptional controls underlying the induced tissue mineralization [27]. Although there are overall reported in vitro osteogenic inductive effects, differences among CSCs, as well as those concerning the same cement, have been noticed regarding cell proliferation, gene expression profile, osteogenic functional parameters, and signaling mechanisms [27]. The wide variability of the in vitro protocols, i.e., regarding the cell model and culture conditions, type of assay (direct/indirect contact), exposure time, and analyzed outcomes, results in evident inconsistencies. Additionally, as a major limitation, these systems lack the 3D cell-to-cell and cell-to-matrix interactions, thus hindering translational predictions [32]. Although there is the more representative contribution of in vivo pre-clinical research, these models are invariably associated with strict ethical considerations and high production costs. 

Accordingly, the research paradigm is changing, which is reflected by the notorious effort and investment to pursue the defined 3Rs (Replacement, Reduction, Refinement) policy by developing new research models, tools, and approaches to minimize animal use and suffering [33,34,35]. In this context, ex vivo organotypic setups are appealing as they surmount some of the constraints faced with in vivo models and may function as transition systems between the in vitro and in vivo research by preserving the spatial and organizational in situ 3D complexity, delivering representative and translational data and being normally cost-effective options [36]. In previous work, the research team reported the suitability of the embryonic chick femur model to address the osteogenic potential of MTA-based endodontic cements [37]. This system has proven to be a useful model in bone-related biology to address development, regeneration, and response to external stimuli [38,39,40,41,42]. In this model, 11th-day embryonic femurs were cultured ex vivo at the air/liquid interface. Subsequently, during the ex vivo culture of about 10–11 days, a timely stage of cell proliferation and steady growth of bone and cartilage occur [38]. Although there are differences in development and growth between avian and human bone, the cellular and signaling events seem to be similar across both species [38].

Putting together some of the pointed shortcomings, this work compared the biological response to the fast-setting cements Biodentine™ (a second-generation material following MTA), and TotalFill^®^ BC RRM™ Fast Putty (a pre-mixed material), with the classical slow-setting ProRoot^®^ MTA, within an ex vivo model of bone development, i.e., the embryonic chick femur model. Ex vivo growing femurs were exposed to the set of cement eluates, and at the end of the culture period, they were evaluated for osteogenesis/bone formation by combining microtomographic analysis and histomorphometric assessment upon selective histochemical staining.

## 2. Materials and Methods

### 2.1. Calcium Silicate-Based Cements and Preparation of the Extracts 

The following cements were tested: Biodentine™ (Septodont, Saint-Maur-des-Fossés, France) and TotalFill^®^ BC RRM™ Fast Putty (FKG Dentaire SA, La Chaux-de-Fonds, Switzerland), as fast-setting CSCs, and ProRoot^®^ MTA (Dentsply DeTrey, Wallenhorst, Germany) as the long-setting reference control material. The chemical compositions, as provided by the manufacturer and batch number, are detailed in Table 1.

The cements were manipulated according to the manufacturers’ instructions, under aseptic conditions. The cement samples were established with 1 mm thickness on a plastic tissue culture coverslip (Æ 13 mm, Sarstedt Inc., Newton, MA, USA) and allowed to set for 24 h, at 37 °C, in a 100% humidified atmosphere.

Cement extracts: The coverslips with the set cements (1 mm thickness circles with Æ 13 mm, 1.33 cm^2^) were placed into sterilized 24-well plates and incubated in basal cell culture medium (0.6 mL/well), i.e., α-minimum essential medium (MEM α, nucleosides, powder, Gibco^®^, Waltham, MA, USA; Ref. 11900073) supplemented with 100 IU/mL penicillin, 100 μg/mL streptomycin, and 2.5 μg/mL amphotericin B (Gibco^®^, Waltham, MA, USA), for 24 h at 37 °C in a humidified atmosphere (5% CO_2_/air). After incubation, the media, hereinafter extracts, were collected, filtered (0.2 μm), and diluted in the basal culture medium. Extracts of 1%, 10%, and 20% were tested in the ex vivo experiments. The undiluted and diluted extracts were assessed for pH. Further, the extraction medium and the 20% extracts were analyzed for calcium ion concentration by direct aspiration into an air–acetylene flame under atomic absorption spectrometry (Varian AA300), using standardized conditions (SMEWW 3111B).

### 2.2. Testing the Cement Extracts in the Ex Vivo Embryonic Chick Femurs Model

Fertilized chick eggs (Gallus domesticus) were used in this study. The experimental use for research of avian fetal forms within the first two-thirds of development is not encompassed by current European (Directive 2010/63/EU) or National (Decreto-Lei *n*.° 113/2013) legislation, precluding the need for regulatory approval of the experimental procedures. Briefly, eggs were incubated in an Octagon 40 ECO rotating egg incubator (Brinsea, Weston-super-Mare, UK), at 37.5 °C and 50% humidity. On day 11, the embryos were euthanized and whole femurs were carefully dissected, removing the soft tissue such as ligaments and adherent muscles while preserving the periosteum. Femurs (*n* = 10 per group) were settled onto a Netwell Insert (440 μm mesh size polyester membrane, 30 mm diameter, Corning Inc., New York, NY, USA) into 6-well tissue culture plates (Costar^®^, Corning Inc., New York, USA) containing 1 mL of culture medium (composition as above), at the air/liquid interface (organotypic culture) and incubated in a humidified atmosphere of 5% CO_2_/37 °C. After 24 h, the medium was removed and the embryonic chick femurs were further cultured for 10 days under control conditions (1 mL of basal culture medium in the absence of the extract) or exposed to the cement extracts (1%, 10%, and 20%, 1 mL) (Supplementary Material – Figure S1). The culture medium was changed every 24 h (control medium/medium containing the extract). At the end of the culture period, the femurs were washed in phosphate-buffered saline (PBS, pH = 7.4), fixed, and processed for microtomography and histological evaluation, using standardized conditions. 

### 2.3. Microtomographic Evaluation 

Femur specimens were imaged in a SkyScan 1276 micro-computed tomography scanner (Bruker, Kontich, Belgium). Sample containers (1.5 mL Eppendorf tubes) were set on the sample stage and imaged using a detector assembly over a 360° sample rotation. Data was acquired under the following settings: source voltage of 40 kV, source current 100 μA, an exposure time of 800 ms, and a voxel size of 4.5 μm. Raw data were reconstructed in the NRecon software v.1.7.4.2, upon correction for beam hardening, ring artifacts, and misalignment. CT Analyzer software v.1.17.7.2 was used to visualize and analyze the reconstructed images for bone volume (BV) and tissue volume (TV). Thresholding was applied to obtain an average binarized grayscale for the reconstructed datasets. Results were expressed as BV/TV (%).

### 2.4. Histological Processing, Histochemical Staining, and Histomorphometric Analysis

Femurs were washed, fixed (4% paraformaldehyde, 24 h), dehydrated in graded alcohols, cleared in Histoclear (National Diagnostics, Hull, UK), and embedded in paraffin in an automated tissue processor (STP 120 Spin Tissue Processor; Thermo Scientific, Waltham, MA, USA). Samples were sectioned (7 μm thickness) with a micrometer (Microm HM 335 E, Thermo Scientific, Waltham, MA, USA), transferred to glass slides, deparaffinized, hydrated, and stained in Alcian blue solution (proteoglycans staining), (1 g Alcian blue, Sigma (St. Louis, MO, USA); 3 mL glacial acetic acid, Fisher; and 97 mL distilled water; pH 2.5; 30 min RT). Afterwards, samples were rinsed in tap water and stained in picrosirius red solution (collagen staining), (0.1 g Sirius red, Aldrich; and 100 mL saturated aqueous picric acid, Sigma; 1 h RT). Samples were also processed for von Kossa staining (mineralized tissue); sections were incubated in a 1% silver nitrate under ultraviolet light for 20 min, rinsed, immersed in 5% sodium thiosulfate for 5 min to remove unreacted silver, and counterstained with nuclear fast red for 5 min. In the end, specimens were dehydrated, cleared, and mounted. 

Histomorphometric analysis. Images were captured using an Axiolab5 microscope and Axiocam208c imaging system (Zeiss, Oberkochen, Germany) (*n* = 10 per condition, with a total of six sections from each femur/histologic stain). Histomorphometric measurements of positive structures in histologic sections were quantified as a proportion of the total image area, based on color thresholds on ImageJ software (version 1.51j8, National Institutes of Health, Bethesda, MD, USA). Picrosirius red staining was further evaluated upon polarized light in the referred microscope and imaging system.

### 2.5. Statistical Analysis

Quantitative data measurements were calculated and presented as mean ± standard deviation. Data normality was determined by the Shapiro–Wilk test. For normal data sets, one-way ANOVA was performed, followed by multiple comparisons using Tukey’s test. For non-parametric data sets, the Kruskal–Wallis test was performed, followed by multiple comparisons using Dunn’s tests. SPSS Statistics (IBM, version 26) was used for calculations. Statistical differences were considered to be significant if *p* values ≤ 0.05.

## 3. Results

The set cement eluates from two fast-setting materials, Biodentine^TM^ and TotalFill^®^, were compared to those from the long-setting ProRoot^®^ MTA, for their effect on the osteogenic tissue response within an ex vivo bone development model. 

The cement extracts were assessed for pH and calcium ion concentration. The undiluted extracts presented a pH~9.5, whereas the tested extracts (1%, 10%, and 20%) showed a pH~7.4. Calcium ion concentration in the basal culture medium (the extraction fluid) was 69.7 ± 5.2 mg/L, and the 20% extracts presented an increment of 9.6 ± 1.2 mg/L (ProRoot^®^ MTA), 20.4 ± 2.8 mg/L (Biodentine^TM^) and 11.2 ± 1.9 mg/L (TotalFill^®^).

Eleven-day-old embryonic chick femurs were cultured in organotypic conditions for 10 days in the absence of extracts (control) and exposed to the cement extracts. Daily observation of the cultured femurs showed that they preserved morphological characteristics and integrity in all conditions. At the end of the 10-day culture period, bone tissue samples were characterized for microtomographic analysis and morphometric assessment of the bone volume ratio, as well as histochemical staining for collagen and proteoglycan components of the extracellular matrix—AB/SR staining, and for mineral deposition—von Kossa staining, and respective histomorphometric indexes.

**Figure 1:**
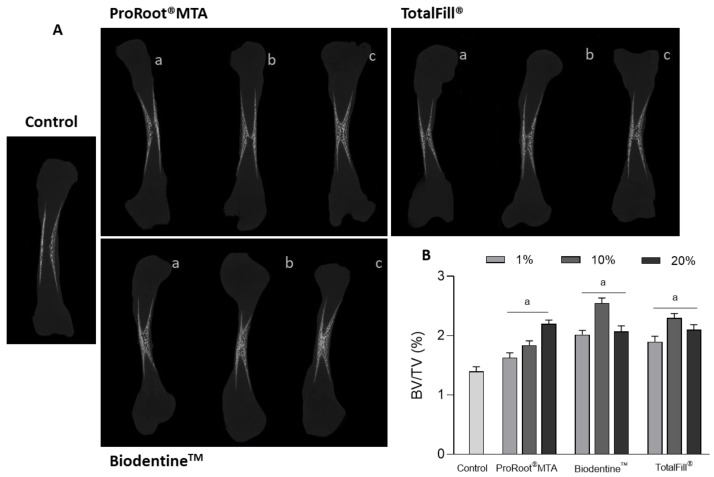
(**A**) Representative microtomographic sections of the embryonic femurs grown ex vivo for 10 days. Samples exposed to 1% (a), 10% (b) or 20% (c) of the diluted extracts. (**B**) Quantitative histomorphometric determination of the bone volume ratio (BV/TV, BV—bone volume, TV—tissue volume); a—significantly different from the control, *p* < 0.05.

Figure 1A,B show, respectively, a set of representative microtomographic images of the coronal section and the morphometric index BV/TV of the femurs. In the control, bone formation was observed with a trabecular organization of the mineralized tissue at the mid-diaphysis, progressing centripetal from the cortical limits into the most internal region of the tissue structure. Comparatively, femurs grown in the presence of the cement extracts revealed an increased deposition of mineralized tissue, with an increased and denser trabecular organization being observed at the central region, with the exposure to Biodentine^TM^ seeming to induce the highest effect, considering the tested dilutions. This was confirmed by the BV/TV index. Control femurs presented the lowest value, while the exposed ones displayed significantly higher values in the tested concentration range. For all cements, the inductive effect was dose-dependent, but different patterns were noticed among the materials. For MTA, the BV/TV index increased gradually with the extract dilution. Thus, compared to control, increments of ~14%, 24%, and 47% were measured after exposure to 1%, 10%, and 20% extracts, respectively. However, for Biodentine^TM^ and TotalFill^®^, BV/TV index was higher, in the range of 1% to 10%, and decreasing after exposure to the 20% extracts (although values were still significantly higher than those of the control). Exposure to 1% and 10% Biodentine^TM^ extracts resulted in an induction of ~36% and 72%, respectively, whereas with TotalFill^®^ extracts values were ~28% and 54% higher, than those of the control. For the 20% extracts, increases were similar for Biodentine^TM^ and TotalFill^®^, compared to the control (~40%). 

**Figure 2:**
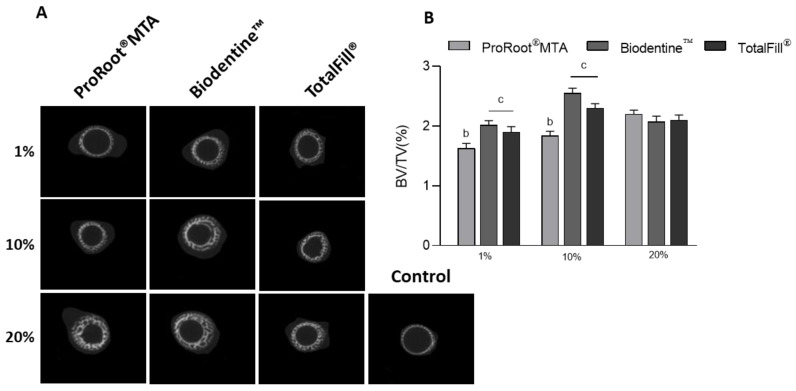
(**A**) Representative microtomographic transversal sections of the embryonic femurs grown ex vivo for 10 days. (**B**) Quantitative histomorphometric determination of the bone volume ratio (BV/TV, BV—bone volume, TV—tissue volume); b—significantly different from c, *p* < 0.05.

Figure 2A displays cross-sectional images from the mid-coronal region of the mineralized diaphysis of control and exposed femurs. Information provided by the images is aligned with that shown in Figure 1A. Figure 2B highlights the difference in the inductive effects caused by the fast-setting cements compared to those observed with ProRoot^®^ MTA, in the tested dilutions. At 1% and 10%, Biodentine^TM^ and TotaFill^®^ extracts caused higher BV/TV index values, with greater induction being achieved in the femurs exposed to the 10% extracts. Of these, Biodentine^TM^ produced the highest BV/TV values. The 20% MTA extract was still stimulatory but, at this concentration, Biodentine^TM^ and TotalFill^®^ extracts caused decreased osteogenic enhancement, as compared to those attained with 1% and 10% extracts.

**Figure 3:**
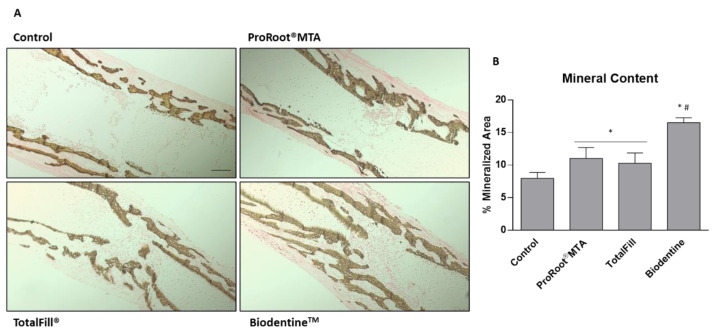
(**A**) Representative tissue sections of the embryonic femurs grown ex vivo for 10 days in the presence of 10% extracts, dyed with von Kossa histochemical stain. (**B**) Quantitative analysis of the von Kossa-stained tissue area, evidencing the mineralized region. * Significantly different from the control; # Significantly different from ProRootMTA and TotalFill, *p* < 0.05.

The observed image data is corroborated by the assessment of von Kossa-stained sections (Figure 3A), showcasing the distribution of the tissue mineral deposition, focusing on exposure to 10% extracts. Comparatively, in the control, the mineralized trabecular structure is limited to the peripheral region of the femur diaphysis, with small but interconnecting trabeculae occupying the marginal region. Cultures exposed to ProRoot^®^ MTA extracts presented a denser trabecular organization, with larger trabeculae growing centripetal towards the central region of the bone. TotalFill^®^ and Biodentine^TM^ presented a more mature trabecular organization, further evidencing an increased mineralized area, with the defined centripetal growth. Qualitative analysis is supported by the morphometric determination of the mineralized tissue area (Figure 3B), evidencing an increase of around 35% for ProRoot^®^ MTA and TotalFill^®^, and around 200% for Biodentine^TM^, compared to the control. Additionally, Biodentine^TM^ values were significantly higher than those regarding ProRoot^®^ MTA and TotalFill^®^.

**Figure 4:**
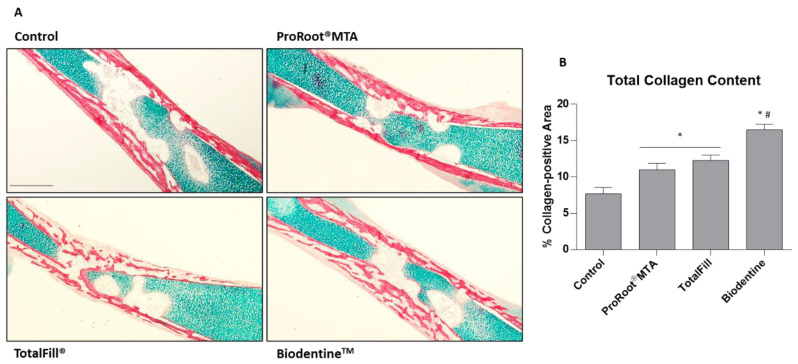
(**A**) Representative tissue sections of the embryonic femurs grown ex vivo for 10 days in the presence of 10% extracts, dyed with Alcian Blue/Sirius Red histochemical stain. (**B**) Quantitative analysis of the Sirius red-stained tissue area, evidencing the collagenous region. * Significantly different from the control; # Significantly different from ProRootMTA and TotalFill, *p* < 0.05.

For a compositional evaluation and morphological organization of the tissue structure, the mid-diaphysis region was further characterized with AB/SR histochemical staining, disclosing, respectively, proteoglycan- and collagen-rich tissue sections, the latter associated with the osteogenic transition of the precursor populations (Figure 4A). Low-magnification micrographs of the mid-diaphysis region showed an intense and marginally distributed collagenous matrix deposition, within the proteoglycan-rich tissue predominating within the central region of the bone. The collagenous tissue evidenced a trabecular arrangement, in accordance with the pattern verified with the von Kossa staining (Figure 3A) and microtomographic analysis (Figure 1 and Figure 2). Femurs exposed to cement extracts presented a similar structure and morphological arrangement, despite the evidence of a more complexly arranged trabecular structure, particularly evident with TotalFill^®^ and Biodentine^TM^ formulations, in which the centripetal progression of the trabecular organization was quite evident. Accordingly, histomorphometric determination of the collagen-positive tissue corroborates the observed findings, with significantly higher levels of about 27%, (ProRoot^®^ MTA), 56% (TotalFill^®^) and 200% (Biodentine^TM^), as compared to the control (Figure 4B). Values for the latter material were much higher than those associated with the other two cements.

**Figure 5:**
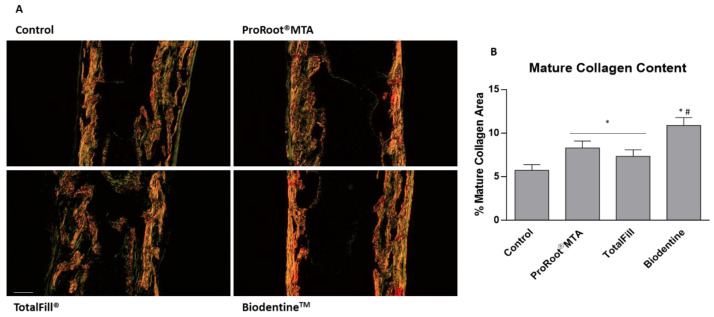
(**A**) Representative tissue sections of the embryonic femurs grown ex vivo for 10 days in the presence of 10% extracts, dyed with Alcian blue/Sirius red histochemical stain, observed under polarized light, highlighting the collagen birefringence. (**B**) Quantitative analysis of the red fluorescent area, evidencing the matured collagenous region. * Significantly different from the control; # Significantly different from ProRootMTA and TotalFill, *p* < 0.05.

Lastly, aiming to disclose the compositional structure and organization of the collagenous matrix, AB/SR-stained sections were characterized upon polarized illumination, allowing to detail of the birefringence of the collagen fibers—intimately associated with the degree of maturation (Figure 5A). In the control, collagen fibers presented a thick and organized structure, with a predominant orange to red coloration—related to a more mature phenotype, with interspersed green fibers, associated with less mature fibers—found to be more abundant at the edge of the trabecular arrangement. A comparable fiber arrangement was observed within tissues exposed to the extracts, despite the increased thickness and color intensity, as confirmed by the quantitative assessment of the mature collagen fibers (orange to red coloration) (Figure 5B). Again, values attained with the Biodentine^TM^ extract were significantly higher than those found with ProRoot^®^ MTA and TotalFill^®^ extracts.

## 4. Discussion

Calcium silicate-based cements are used in a variety of clinical conditions relying on their inductive effect on tissue mineralization. The present work compared the osteogenic/bone formation potential of two fast-setting cements, Biodentine^TM^ and TotalFill^®^, with the gold standard and long-setting ProRoot^®^ MTA, using the 3D organotypic embryonic chick femur development model. This study aimed to take a step forward on the functionality of these cements compared with previous work, showing that the formulations were not cytotoxic to human fibroblasts in an indirect contact assay as established by ISO 0993-1:2018 guidelines and were also devoid of irritation potential in the in vivo chorioallantoic membrane (CAM) assay [43]. Further, performing this ex vivo translational approach allowed us to explore the suitability of this model as an alternative research framework, intended to advance the 3R’s policy [33,34].

The three CSCs differ in the physicochemical profile, reflected in distinct handling properties, the structure of the unset and set material, the kinetics of the eluates’ release, and the setting time—all clinically relevant features [17,21,44]. Nevertheless, owing to the similar calcium silicate base composition, they share most of the clinical indications [1,3,6]. However, the comparative clinical outcomes between fast- and slow-setting cements show variable and inconsistent results, particularly those involving TotalFill^®^, the least studied of the three cements [6,45]. Further, the same concerns exist regarding the understanding of the underlying cellular responses and associated mechanisms, which have been mostly addressed by in vitro studies that involve a simplified non-representative two-dimensional environment surrounding the bone cells and a multiplicity of experimental protocols [27,46,47,48]. The use of ex vivo organotypic models of bone development represents a step forward complementing preclinical evaluation [32,49], potentially aiding in establishing the role of the newer CSCs in reparative endodontics.

The embryonic chick model was previously established following a detailed characterization of the ex vivo bone development and formation events in different embryonic developmental stages and a variety of experimental conditions, i.e., in the absence/presence of osteogenic and chondrogenic conditions [39,50]. The embryonic 11th-day was proven to be the most appropriate to set up the ex vivo organotypic culture. At this stage, the skeletal differentiation and bone mineralization had just started, and the ex vivo growing femur kept the tissue integrity during the subsequent 10–11 days of culture, showing a timely bone development [50]. Additionally, the model is appropriate to study bone formation as the earlier staged femurs are programmed to drive the bone formation process rather than bone remodeling [50]. The culture conditions are quite simple involving only a standard culture medium without fetal bovine serum supplementation, as it is normally required for cell cultures, thus avoiding the differences in the batch and commercial sources, providing better correlation between studies. This ex vivo model has been used to address a large variety of bone-related setups [37,40,41,42,51,52].

In this work, the 11-day-old embryonic femurs were grown ex vivo for 10 days in the presence of the cement extracts. The undiluted extracts were prepared from the set materials using a basal control medium as the extraction solution, which was further diluted for the exposure experiments (1%, 10%, and 20% extracts). The undiluted extracts showed a pH~9.5, thus exhibiting the alkalinizing effect normally described for these formulations [53,54]. However, due to the buffering effect of the medium, the pH of the diluted extracts was kept at ~7.4, similar to that of the extracellular fluid. The extraction medium contained a variety of amino acids, vitamins, ribonucleosides, deoxyribonucleosides, D-glucose, phenol red (the pH indicator), and sodium pyruvate. Further, the inorganic salts include calcium chloride, magnesium sulfate, potassium chloride, sodium chloride, and sodium phosphate monobasic as the buffer, keeping the pH ~7.4. This extraction fluid is a standard synthetic medium routinely used in cell culture experiments having the appropriate organic composition, pH, and osmolality to support the survival, growth, and interaction of mammalian cells [55,56], thus being also used for the ex vivo growth of the embryonic femurs.

Calcium ions are the major elements that leach out from the set CSCs [28]. Ca^2+^ is released as a by-product of the hydration reaction having a key role in the inductive osteogenic potential of these materials [28,57]. As such, the concentration of these ions was evaluated in the extracts (20% extract). Ca^2+^ concentration in the basal culture medium (the extraction fluid) was around 69 mg/L, a value similar to that listed in the datasheet of the medium (72 mg/L). After the 24 extraction period, Ca^2+^ levels increased in the extraction medium. The increment was similar in ProRoot^®^ MTA and TotalFill^®^ extracts and lower than that measured in the Biodentine^TM^ extracts (about half of the values). Comparison with the reported literature data is neither easy nor rigorous due to the wide heterogeneity in extraction protocols. It is worth noting that normally the used extraction vehicles are phosphate-containing and calcium-deprived fluids, thus the release of calcium ions becomes the limiting parameter [57]. Nevertheless, consistently, Biodentine^TM^ accounts for a higher Ca^2+^ release compared to that from ProRoot^®^ MTA, in a variety of extraction settings [44,57,58,59,60] but few studies have shown similar or even lower values according to the experimental design [61]. Variable results have been described for the Ca^2+^ levels in TotalFill^®^ extracts, but some studies reported higher values compared to that in the ProRoot^®^ MTA extracts [61]. Inconsistencies also occur regarding Ca^2+^ levels released from TotalFill^®^ and Biodentine^TM^ [28,62]. Although Ca^2+^ leaching is considered to be an important parameter in the osteogenic performance of CSCs, no evidence regarding the appropriate/desirable calcium levels and/or release pattern is available [63]. Therefore, materials with different Ca^2+^ leaching profiles are usually evaluated using various cyto- and biocompatibility assays aiming to assess a dose–response relationship between Ca^2+^ release/levels and biological response. However, a quantitative correlation path is yet to be determined and, of most relevance, in vitro values do not reflect the clinical scenario [64].

In the present work, the three cement extracts increased the ex vivo osteogenesis in a dose-dependent way, although different patterns and mineralization indexes were seen with the fast- and slow-setting materials, as assessed by histomorphometric indexes of the microtomographic (BV/TV, %) and histochemical (% of the mineralized area; % of collagen area; % mature collagen area) analysis. The highest values were observed with Biodentine^TM^ and TotalFill^®^ following exposure to the 10% extracts, with the 20% extracts, comparatively, inducing a decreased outcome, despite being significantly higher than that attained in the control. Comparatively, ProRoot^®^ MTA extracts caused lower inductive effects (1% and 10% extracts) and values increased with the extract concentration. It appears that, at least partially, some sort of correlation might be established between calcium ion levels and the observed inductive effects, i.e., Biodentine^TM^ extracts showed the highest [Ca^2+^] and were associated with the higher osteogenic indexes. As the effect of Ca^2+^ is dose-dependent [28], it might be hypothesized that levels of these ions in the 20% extract might already induce, comparatively, a decreased osteogenic outcome, exceeding the range of maximal osteogenic induction. However, in the used protocol, TotalFill^®^ extracts displayed similar Ca^2+^ levels as those of ProRoot^®^ MTA extracts, but the 10% extract caused significantly higher osteogenic induction than the corresponding MTA extract, and the 20% extract comparatively induced a reduced osteogenic stimulation, unlike the 20% MTA extract. Thus, similar to that referred to previously, a quantitative correlation between Ca^2+^ levels in cement eluates and the attained inductive effects were not established. This is expected considering the differences in the physicochemical profiles of the three cements that, would certainly deliver eluates with a diverse composition. Although the cements are all calcium silicate-based formulations, they include different additives used to improve the cements’ handling and performance. Accordingly, a complex combination of inert, inhibitory and inductive molecules with distinct release kinetics would be present in the extracts. Considering the tested cements, it has been reported that bismuth oxide, in the ProRoot^®^ MTA formulation, appears to be associated with some toxicity concerns [65]. Biodentine^TM^ seemed to overcome this limitation, as zirconium oxide, used as a radiopacifier agent, seems to be inert. Nonetheless, the inclusion of a setting accelerator (calcium chloride) and a water-soluble polymer may significantly affect the release features due to the reaction with water demands [13]. On the other hand, compared to ProRoot^®^ MTA and Biodentine^TM^, the inclusion of monobasic calcium phosphate (a second cementitious phase) in the TotalFill^®^ formulation might influence the hydration of the material and calcium hydroxide release, as reported in a study conducted with a biphasic experimental cement [66]. This phase was incorporated with the aim of enhancement of physical, chemical and biological properties, but data on its real contributions are limited and controversial [27].

In regard to the experimental model, it is worth considering that the assayed ex vivo system may present phylogenetically related biological differences, given the dissimilarities between the diapsid (birds) and synapsid (mammals) lineages. In addition, it does not replicate the in vivo microenvironment in which the dynamic interstitial fluid and blood flow conjoin to modulate local concentrations of CSC leachables, as it is also devoid of further interactions with distinctive cellular and molecular elements—such as those from the immune-inflammatory response, or other systemic influences—that can further modify the biological outcomes. Notwithstanding, overall, the results regarding the ex vivo osteogenic potential of the three tested calcium silicate-based cements are in line with the reported data from the literature. To date, in vitro and laboratory studies accounted for positive features regarding the physicochemical and biological properties of these types of materials, although high variability and sometimes inconsistency are described, mostly associated with the heterogeneity of methodologies [20,67]. Using the same experimental protocol, the potential to induce osteogenesis appears to be diverse among the cements, as they seemed to induce different phases of cellular differentiation [68]. Additionally, the available clinical data support a positive outcome of the CSCs [19,69,70], namely the tested formulations. Compared to the “gold standard” ProRoot^®^ MTA, Biodentine^TM^ has also been thoroughly evaluated, accounting for similar or even higher positive outcomes being suggested for similar clinical conditions [71,72]. Fewer clinical studies/trials are available regarding the pre-mixed materials, namely TotalFill^®^. Still, as happens with the other cements in this group, positive results have also been described [69,71]. This highlights two aspects, i.e., the overall performance of the CSCs as a group and/or the need for further fundamental and clinical assessment, validating efficacy in specific clinical applications.

## 5. Conclusions

The fast-setting Biodentine^TM^ and TotalFill^®^ cements were compared with the slow-setting ProRoot^®^ MTA regarding the ex vivo osteogenic potential in an organotypic bone developmental bone, using an indirect contact assay. ProRoot^®^ MTA and TotalFill^®^ extracts presented similar levels of calcium ions, but significantly lower than those released from Biodentine^TM^. All cements increased the osteogenesis/tissue mineralization microtomographic (BV/TV) and histomorphometric (% of mineralized area; % mature collagen area) indexes, although displaying distinct dose-dependent patterns and quantitative values. Biodentine^TM^ presented the best performance. Results accounted for the suitability of the ex vivo chick embryonic femur model to address the osteogenesis/tissue mineralization potential of these formulations, fueling representative translational research.

## Figures and Tables

**Table 1:** Calcium silicate-based cements used and respective composition, manufacturer, and batch number.

Material	Composition	Manufacturer	Batch No.
ProRoot^®^ MTA	Tricalcium silicate, dicalcium silicate, tricalcium aluminate, and calcium sulfate.	Dentsply DeTrey GmbH, Germany	0000301574
Biodentine^TM^	Powder: tricalcium silicate, dicalcium silicate, calcium carbonate, iron oxide, zirconium oxide. Liquid: water with calcium chloride and soluble polymer (polycarboxylate).	Septodont, France	B27532
TotalFill^®^ BC RRMTM Fast Putty	Zirconium oxide, tantalum oxide, calcium silicate, calcium phosphate monobasic and fillers.	FKG Dentaire SA, Switzerland	2100004308

## Data Availability

The data presented in this study are available on request from the corresponding author.

## Supplementary Materials

The following supporting information can be downloaded at: https://www.mdpi.com/article/10.3390/jfb14060314/s1, Figure S1: Schematic representation of the experimental setup.
