# Supplementary figures and images for: Ex Vivo Osteogenesis Induced by Calcium Silicate-Based Cement Extracts
Source: J Funct Biomater. 2023 Jun 7;14(6):314. doi: 10.3390/jfb14060314 (PMC10299110; doi:10.3390/jfb14060314)

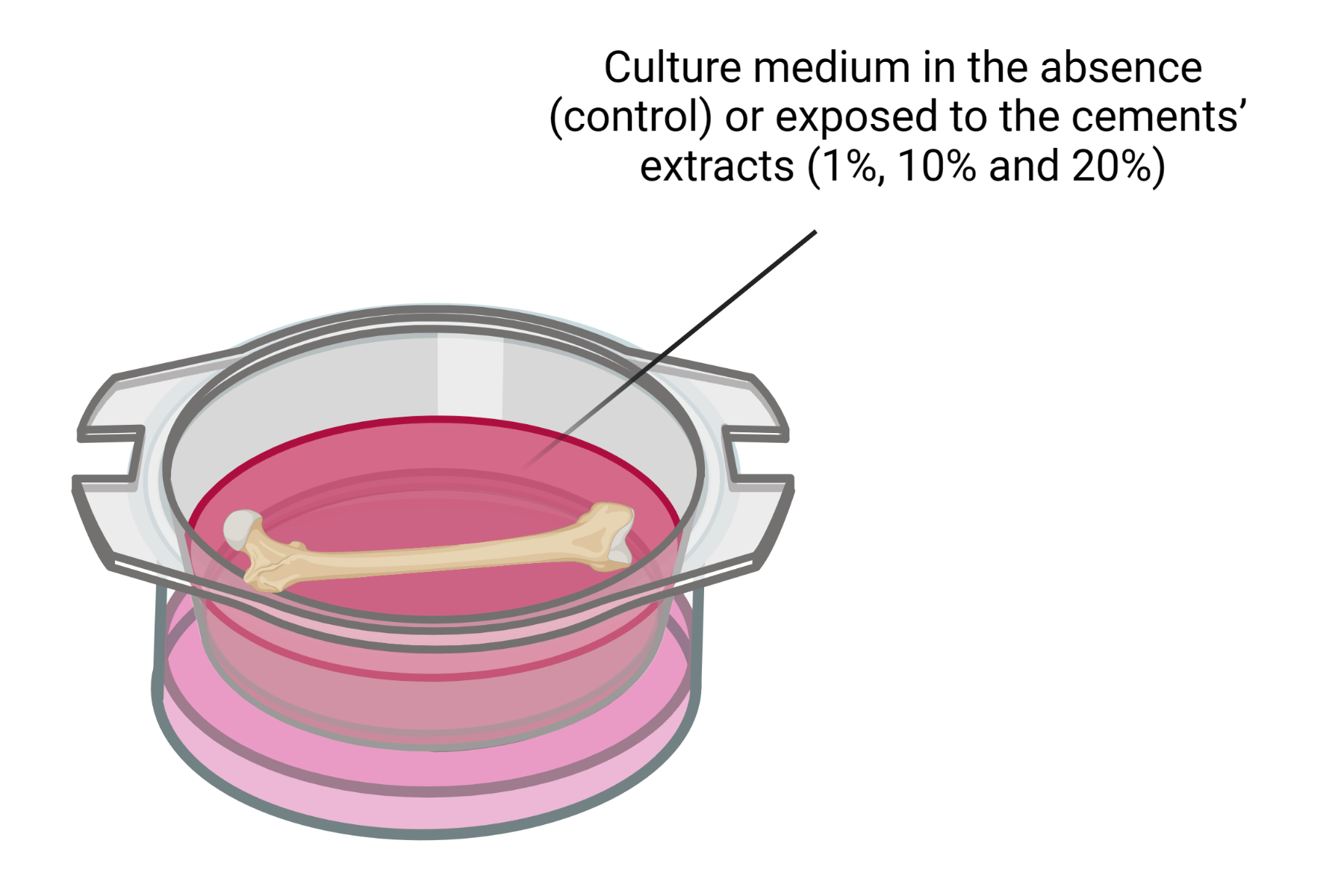

Supplement: Supplementary file 1 [file jfb-14-00314-s001.zip › Figure S1.png]
